# An Electrical Capacitance Array for Imaging of Water Leakage inside Insulating Slabs with Porous Cells †

**DOI:** 10.3390/s19112514

**Published:** 2019-06-01

**Authors:** Rui Li, Yi Li, Lihui Peng

**Affiliations:** 1 Department of Automation, Tsinghua University, Beijing 100084, China; lirui0929@sina.com; 2 Graduate School at Shenzhen, Tsinghua University, Shenzhen 518055, China; liyi@sz.tsinghua.edu.cn

**Keywords:** electrical capacitance array, water leakage imaging, sensitivity map, image reconstruction, capacitance measurement circuit

## Abstract

The paper proposes a capacitance-sensor-array-based imaging system to detect water leakage inside insulating slabs with porous cells, such as anechoic acoustic rubber tiles. The modeling is conducted by using the finite element method to obtain the electrical potential distribution and sensitivity map with the proposed capacitance sensor array. An experimental test setup, which is composed of an eight-electrode capacitance sensor array and a commercialized capacitance bridge instrument for measurement, is developed. Experiments regarding different leakage scenarios are carried out by using the test setup. Preliminary results standing for different water leakage cases, which are based on the experimental data obtained from the test setup, are presented and depicted as images reconstructed by using different algorithms including the linear back projection (LBP), the projected Landweber iteration, and the total variation regularization. These results demonstrate that the proposed capacitance sensor array is feasible and has a great potential for imaging of water leakage inside insulating slabs with porous cells. A cost-effective capacitance measurement circuit for practical applications is also proposed and simulated.

## 1. Introduction

To enhance submarine stealth, most modern submarine hulls are covered with anechoic acoustic rubber tiles. The anechoic acoustic rubber tiles usually have a structure with porous cells inside, which are capable of absorbing active sonar signals from enemy vessels for detecting purposes, meanwhile preventing acoustic noise from the submarine interior, which can be detected by passive sonar, from being transmitted outside [1].

Anechoic acoustic rubber tiles are bonded with the submarine hull by using adhesive. The debonding or detachment of rubber tiles from the steel hull may lead to water leakage into the porous cells, which will absorb less acoustic signals passing through and degenerate the rubber tiles’ acoustic performance. For this reason, one daily routine submarine maintenance task is to inspect whether water has already leaked into the porous cells inside the anechoic acoustic rubber tiles, particularly in the early stage of water permeating due to adhesive debonding.

A number of studies exploring the damping behavior and acoustic performance with acoustic tiles by using numerical analyses and experiments [2,3,4] have been reported. However, fewer literatures on the detection of water permeating into porous cells inside acoustic tiles can be found. On the other hand, nondestructive test methods based on different sensing techniques including X-ray [5], microwave [6,7,8,9], and ultrasonic [10,11,12,13], have been developed for water content detection with different applications. X-ray-based imaging method measures radiation attenuation due to the density differences of objects inside and obtains the image of the structure to be examined, but the measurement system often has a large occupation size, which is not convenient for large samples to be inspected. Microwave-based method is sensitive to water content, but is easily susceptible to electromagnetic interference, particularly while the sensors have to be used and exposed in open environments. Ultrasonic-based method may have a problem with efficiency while used for the water content detection inside objects of huge size. These aforementioned techniques are unsuitable or inconvenient for routine maintenance use for the in situ detection of water leakage into porous cells inside anechoic acoustic rubber tiles. On the other hand, the capacitance sensor, for its cost-effectiveness, simplicity, and nonintrusiveness property, has been widely studied and used for different measurement applications related to permittivity changes [14,15,16,17,18,19]. Tsamis and Avaritsiotis introduced a planar capacitive sensor for monitoring water content in a product line [20]. Ong et al. reported a method of using capacitance to monitor water content in civil engineering materials [21]. Particularly, by using a multielectrode sensor, electrical capacitance tomography (ECT) is imaging and visualizing dielectric changes of multiphase or multicomponent processes with different permittivities [22,23,24,25,26,27,28,29,30,31]. The core of ECT is to arrange a number of electrodes around the object and measure the variations of capacitances between different electrode pairs, and then generate its cross-sectional image by using certain reconstruction algorithms. The variations of capacitance between different electrode pairs are due to the change of permittivity inside the object to be inspected. For different applications, different ECT sensors have been developed, for example, square sensor [32], concentric-annulus sensor with inner and outer electrodes [33], miniature sensor [34], planar sensor array [20], and so forth.

The water leakage into the porous cells of anechoic tiles will change the equivalent permittivity of the medium inside, which may be detectable by appropriately arranging the capacitance electrode array around. Based on this principle, we have proposed and conducted the preliminary modeling of a capacitance array for water leakage detecting and imaging insulating slabs with porous cells inside such as anechoic tiles, which has a great potential to be developed as a test facility for daily routine inspection and maintenance purpose [35].

In this paper, we refine the modeling work to characterize the sensitivity distribution inside the capacitance array proposed in [35] for the detection of water leakage inside the insulating slabs with porous cells such as anechoic rubber tiles. In addition, a prototype experimental test setup that is based on a commercialized capacitance measurement bridge instrument is developed. A series of experiments are carried out by using the test setup and the corresponding experimental results are presented. The paper is organized accordingly: Section 2 describes the basic principle of our proposed capacitance sensor array for water leakage detecting and imaging inside insulating slabs with porous cells and demonstrates the modeling of the electrical potential distribution and sensitivity map with the sensor array. Section 3 introduces the development of the experiment test setup used in this study and presents the preliminary experimental results. Section 4 discusses the difference between the experimental results and the simulation data based results published before in [35], and demonstrates the design of measurement circuit. Finally, the conclusions are drawn in Section 5.

## 2. Working Principle and Modeling of Electrical Capacitance Array

One challenge for the detection of water leakage inside insulating slabs, such as anechoic acoustic tiles having a structure with porous cells, is that leakage usually starts and happens near the adhesive layer for bonding, which is located between the metal wall layer and tile layer. Thus, the detection system can only access and measure from the outside of the tiles. To fulfill this special requirement, we have proposed a capacitance sensor by appropriate arrangement of an electrode array [35].

**Figure 1:**
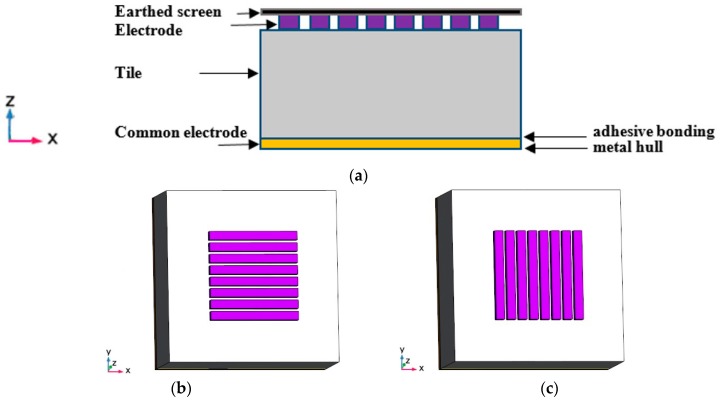
Principle of proposed capacitance sensor array. (**a**) Front view, (**b**) top view of horizontally placed capacitance array, (**c**) top view of vertically placed capacitance array.

Figure 1 depicts the working principle of our proposed electrical capacitance array for water leakage detection and imaging inside slabs with porous cells such as anechoic acoustic tiles. An eight-electrode capacitance sensor array is located horizontally on the outside of the tile at first. Each electrode will form a capacitor with the metal hull used as the common electrode. The capacitance value between each electrode of the capacitance sensor array and the common electrode depends on the permittivity of material between them. While water leakage happens, the quantity of water permeating into porous cells will lead to the equivalent permittivity change and affect the values of these capacitances, which are measured by the electronic measurement hardware system. By rotating the sensor array 90° and adjusting the orientation of the sensor array to the vertical direction, all capacitances between each electrode and the common electrode are measured once again. After all possible capacitances from horizontally and vertically placed sensor arrays are obtained, the permittivity distribution inside the slab can be reconstructed by using certain mathematic methods and demonstrate whether water leakage is occurring.

**Figure 2:**
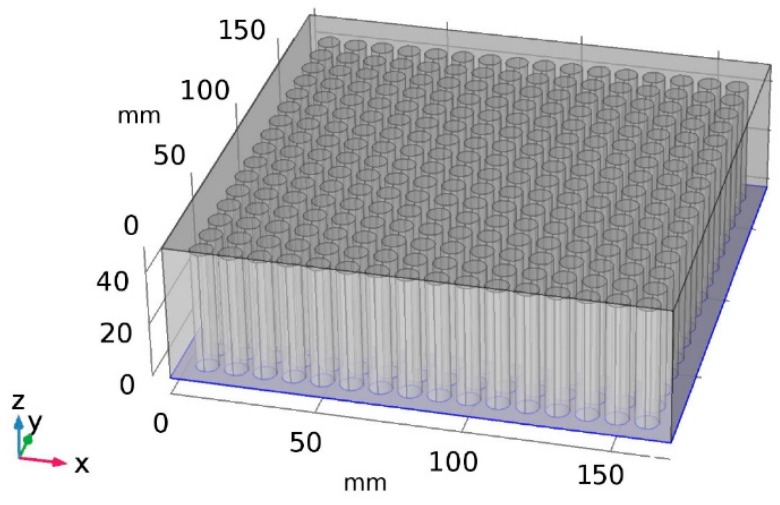
The plexiglass slab used to simulate tile with porous cells inside.

In [35], we have modeled the electrical field and sensitivity distribution in a plexiglass slab with coarse column holes inside. To fulfill the possible practical applications, a plexiglass slab with the structure shown in Figure 2 is used to simulate an anechoic acoustic tile and the characterization of related electrical capacitance sensor array is explored. The slab is a cubic of 170 by 170 by 50 mm, which is the same size as in a previous work [35]. But compared with the previous modeling work in [35], the number of column holes inside the slab to simulate porous cells increases significantly to 16 by 16. The diameter of the column is 8 mm, which is similar to the porous cell size reported in [35]. An eight-electrode array is taken as an example, in which the electrode width is 6 mm and the gap between two adjacent electrodes is 2 mm.

**Figure 3:**
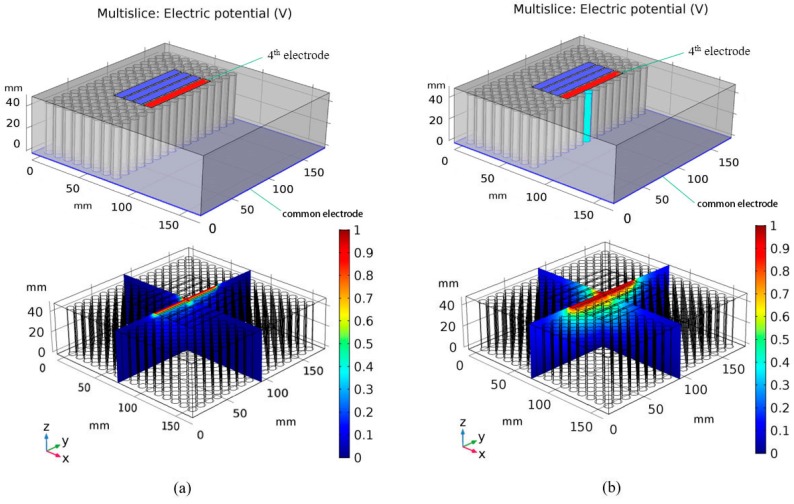
Electric potential distribution while the fourth electrode is activated. (**a**) All column holes have no water inside, (**b**) the central column hole is full of water.

By applying a voltage on one electrode of the array sequentially and connecting other electrodes and the common electrode to ground potential, the electrical potential distribution inside the plexiglass slab shown in Figure 2, which is with fine column holes inside, can be obtained by solving the Laplace equation using the finite element method described in [35]. This procedure is carried out in the commercialized software COMSOLTM. Figure 3 depicts the electric potential distribution inside the slab while the fourth electrode is activated when the central column hole right below the fourth electrode has no water, and when it is full of water. The water quantity in the central column hole is demonstrated in Figure 3 using a sectional cutaway view.

Besides the electrical field, the sensitivity map inside the slab, which describes the relationship between permittivity change and capacitance, is very helpful to characterize the penetration depth of the sensor array. Furthermore, to obtain and illustrate the permittivity distribution inside the slab, the image reconstruction with the proposed sensor array will be carried out after all possible capacitances between each electrode and the common electrode are obtained. This is very similar to image reconstruction in electrical capacitance tomography, which has been widely studied in the past two decades [25,30,36,37,38,39,40,41,42,43]. The sensitivity map also plays an important role in image reconstruction with the capacitance sensor array. The sensitivity $J_{ij}(\sigma )$ at σ(x,y,z) in imaging region, which is with respect to the capacitance change between the ith electrode and the jth electrode, is calculated as [44,45]

(1) $$J_{ij}(\sigma )=-\frac{\int_{\sigma (x,y,z)}\nabla \phi_{i}(x,y,z)\cdot \nabla \phi_{j}(x,y,z)dxdydz}{V^{2}}$$

where $\phi_{i}(x,y,z)$ and $\phi_{j}(x,y,z)$ are the electric potential distribution when the *i*th and the *j*th electrode are excited with voltage V and other electrodes are grounded, respectively.

**Figure 4:**
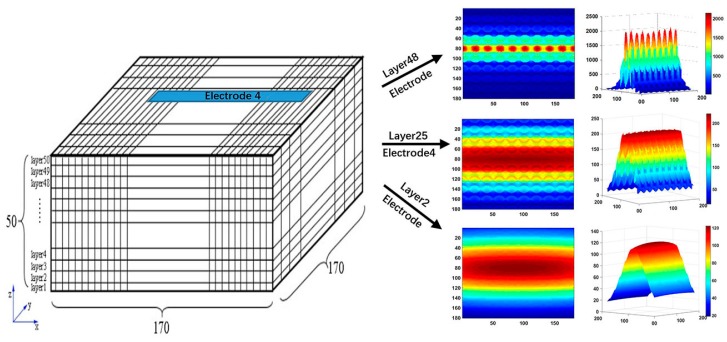
Sensitivity distribution at different *z* directional layers while capacitance array is placed horizontally.

**Figure 5:**
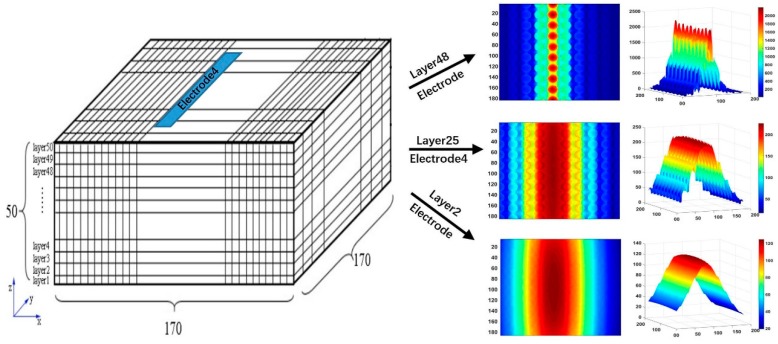
Sensitivity distribution at different *z* directional layers while capacitance array is placed vertically.

**Figure 6:**
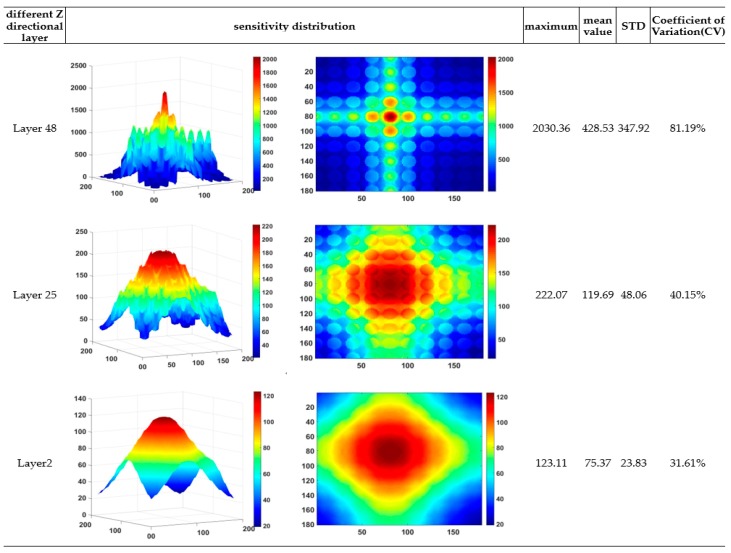
Capacitance array sensitivity distribution in *x–y* plane at different *z* directional layers.

Figure 4, Figure 5 and Figure 6 depict the voxel mesh grid in the imaging area of the capacitance array and illustrate the sensitivity distribution between the fourth electrode and the common electrode in *x*–*y* planes at different *z* locations. In our study, the mesh grid is 170 × 170 × 50.

Compared with those reported in our previous study [35], it is found from Figure 4, Figure 5 and Figure 6 that the sensitivity map becomes more complex while the number of column holes inside the slab to simulate porous cells increases. The sensitivity map shows a multipeak distribution. The high sensitivity peak is located in the cross-region between the horizontal electrode and vertical electrode. Furthermore, the sensor array is more sensitive to the permittivity changes in the column holes than the permittivity changes in other regions. To evaluate quantitatively, maximum, mean, and standard deviation of the sensitivity map at different *z* directional layers can also be found in Figure 6. To compare relatively the homogeneity of the sensitivity map at different *z* directional layers, the coefficient of variation (CV), defined as the ratio of the standard deviation to the mean, is also calculated and provided in Figure 6. It is found from these quantitative criteria that the maximum sensitivity on the top layer near the sensor array electrode is about 20 times that on the layer near the adhesive bonding. Meanwhile, the coefficient of variation decreases significantly from the top layer near the sensor array electrode to the layer near the adhesive bonding, that is, the sensitivity map near the adhesive bonding layer is more homogeneous than that near the sensor array electrode, which means it is much harder to distinguish the exact leakage location in the region near the adhesive bonding.

After the sensitivity map is obtained, image reconstruction algorithms widely used in electrical capacitance tomography, such as the linear back projection (LBP) [25,38], the Landweber iteration [37,38,39], and the total variation-based iteration [41,42,43], can be applied.

The related image reconstruction model can be written as [38]

(2) λ=Sg

where λ stands for the normalized capacitance vector, which is composed of all capacitances between each small electrode of the sensor array and the common electrode. S is the sensitivity matrix, which is calculated according to Equation (1) and depicted in Figure 6. g is normalized permittivity vector, which stands for the permittivity of each voxel and can be visualized as image intensity.

The expression of LBP is as [25,38]

(3) $$\hat{g}=\frac{S^{T}\lambda }{S^{T}u}$$

where $\hat{g}$ stands for the reconstructed permittivity distribution.u is the vector with all elements being 1.

The projected Landweber iteration algorithm is described as [37,38,39]:

(4) $${\hat{g}}_{k+1}=P[{\hat{g}}_{k}-\alpha S^{T}(S{\hat{g}}_{k}-\lambda )]$$

where *P* is the operator in Equation (5), which makes the solution projected to the range [0,1] after each iteration.

(5) $$P[f(x)]=\left\{ \begin{array}{l} 0\;\mathrm{if}\;f(x)<0\\ f(x)\;\mathrm{if}\;0\leq f(x)\leq 1\\ 1\;\mathrm{if}\;f(x)>1 \end{array}\right.$$

The total variation (TV)-based algorithm uses the gradient of permittivity distribution as regularization function and can be described as [41,42,43]:

(6) $$\hat{g}=\arg\;\min\frac{1}{2}{\|Sg-\lambda \|}_{2}^{2}+\mu \int_{\Omega }\left|\nabla g\right|d\Omega$$

where Ω is the imaging region.

## 3. Results

**Figure 7:**
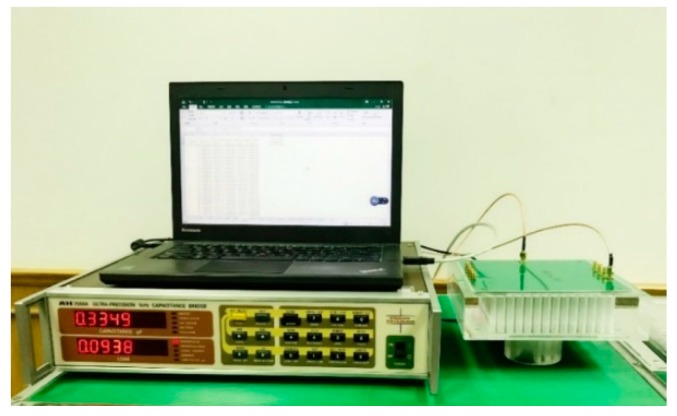
The experimental setup.

To verify the feasibility of the proposed capacitance sensor array for water leakage detection and imaging inside the slab, the related experiments are carried out. The experiment setup as depicted in Figure 7 is composed of an eight-electrode capacitance sensor array, an AH-2550A high-precision capacitance bridge manufactured by Andeen–Hagerling^TM^ (Cleveland, OH, USA) for capacitance measuring, and a Lenovo^TM^ T440 laptop computer for image reconstruction. The capacitance array sensor is fabricated on a two-layer PCB, the layers being the electrode layer and the layer functioning as shielding.

**Figure 8:**
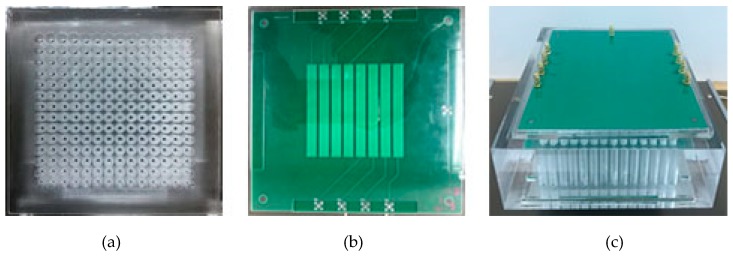
Plexiglass slab and capacitance sensor array used in experiment. (**a**) Top view of plexiglass slab, (**b**) top view of capacitance sensor array, (**c**) plexiglass slab covered with sensor array.

Figure 8 depicts the plexiglass slab and capacitance sensor array used in the experiment. The size of the slab is 170 × 170 × 50 mm. The diameter of the column hole is 8 mm. The size of the electrode array is 90 × 90 mm. The width of the electrodes is 6 mm. The gap between two adjacent electrodes is 2 mm.

Table 1 lists capacitances between different electrode pairs while the four column holes in the central part of the slab are full of water in different quantities, which are measured by using the AH-2550A capacitance bridge. The table provides also the capacitances while all column holes have no water inside and the capacitances while all column holes are 100% full of water. By comparing these capacitances in Table 1, it is found that the capacitance between the fourth electrode and common electrode, and the capacitance between the fifth electrode and common electrode, have a significant change with the variation of water quantity inside the central four column holes, which is consistent with the preset experiment.

**Figure 9:**
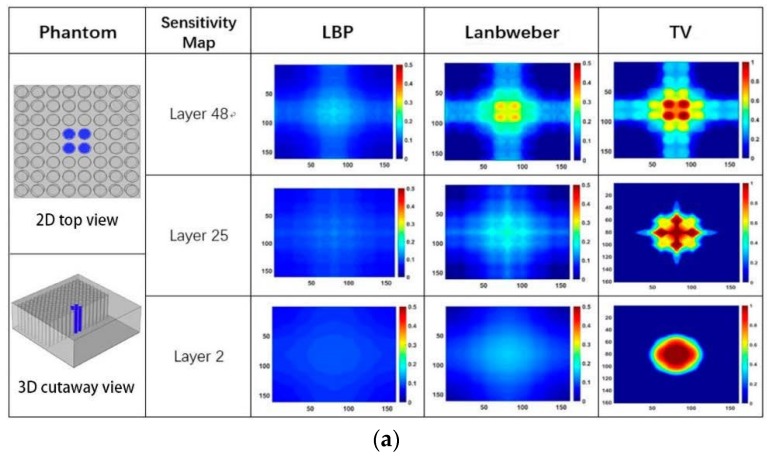

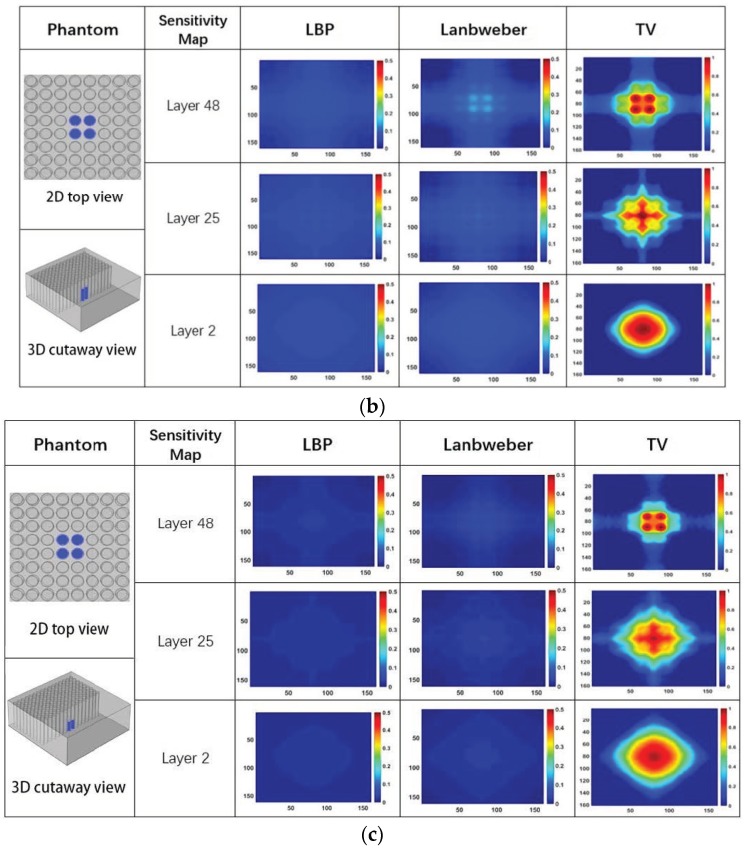
The imaging results while the central four column holes are full of different quantities of water, (**a**) 100% full of water, (**b**) 50% full of water, (**c**) 25% full of water.

Figure 9 shows the images reconstructed from the measured capacitances data in Table 1 by using the LBP, the Landweber iteration, and the total variation-based iteration. The phantom is illustrated by using 2D top view and 3D cutaway view to make it easy to understand. It is found from these reconstructed images that the total variation-based iteration is capable of providing a reasonable but qualitative imaging of water leakage.

**Figure 10:**
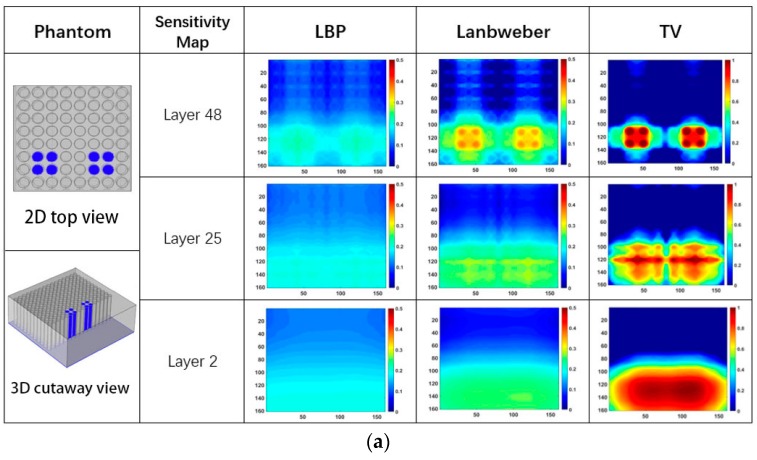

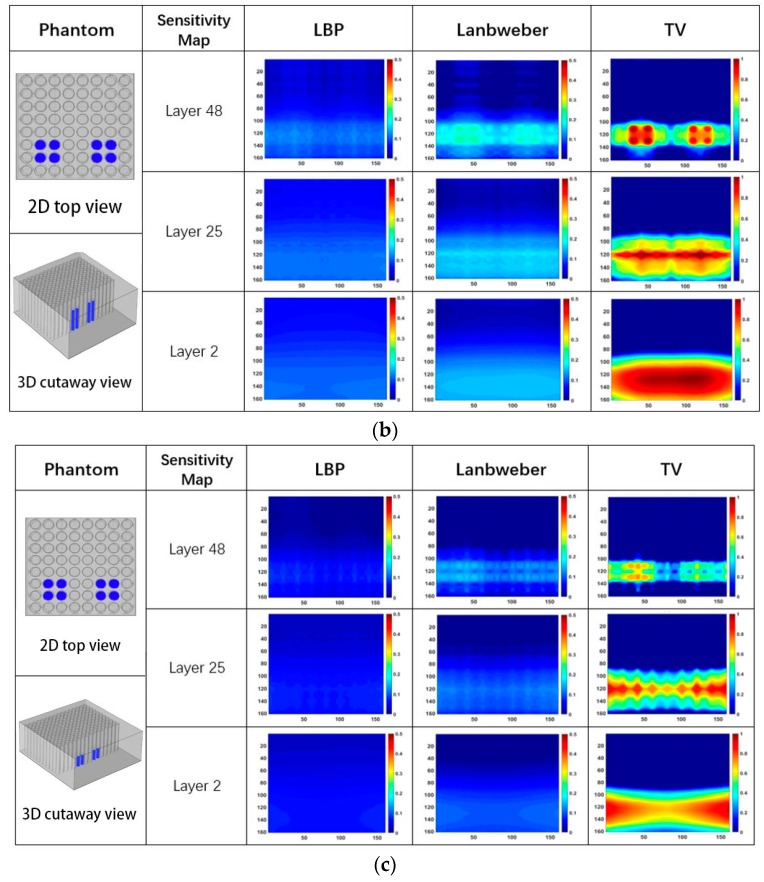
The imaging results while two groups of column holes on one side are full of different quantities of water, (**a**) 100% full of water, (**b**) 50% full of water, (**c**) 25% full of water.

Table 2 lists capacitances between different electrode pairs while two groups of column holes at two different places on one side of the slab are full of different quantities of water. Each group is composed of four column holes. Figure 10 shows the reconstructed images using measured capacitance data in Table 2.

It is found from the results in both Figure 9 and Figure 10 that reconstructed images give larger contrast differences between the position of the water leakage and other areas without water leakage, which demonstrates that the imaging results can clearly reflect the position where water leakage happens. On the other hand, although the results in Figure 9 and Figure 10 can reveal that different quantities of water leakage give different reconstructed images, the differences between the images related to different quantities of water leakage are not significant quantitatively. By comparing the reconstructed images from different layers of the sensitivity map, it is found that discriminating between different quantities of water leakage becomes harder and harder when the sensitivity map layer near the adhesive bonding is used. In addition, the column holes that were 100% full of water generate higher contrast images by adopting the 48th layer sensitivity map near the electrode array. This characterization can be explained intuitively by using the sensitivity map distribution in Figure 4, Figure 5 and Figure 6, which clearly shows that the closer the position to the capacitance sensor array, the higher the related sensitivity.

**Figure 11:**
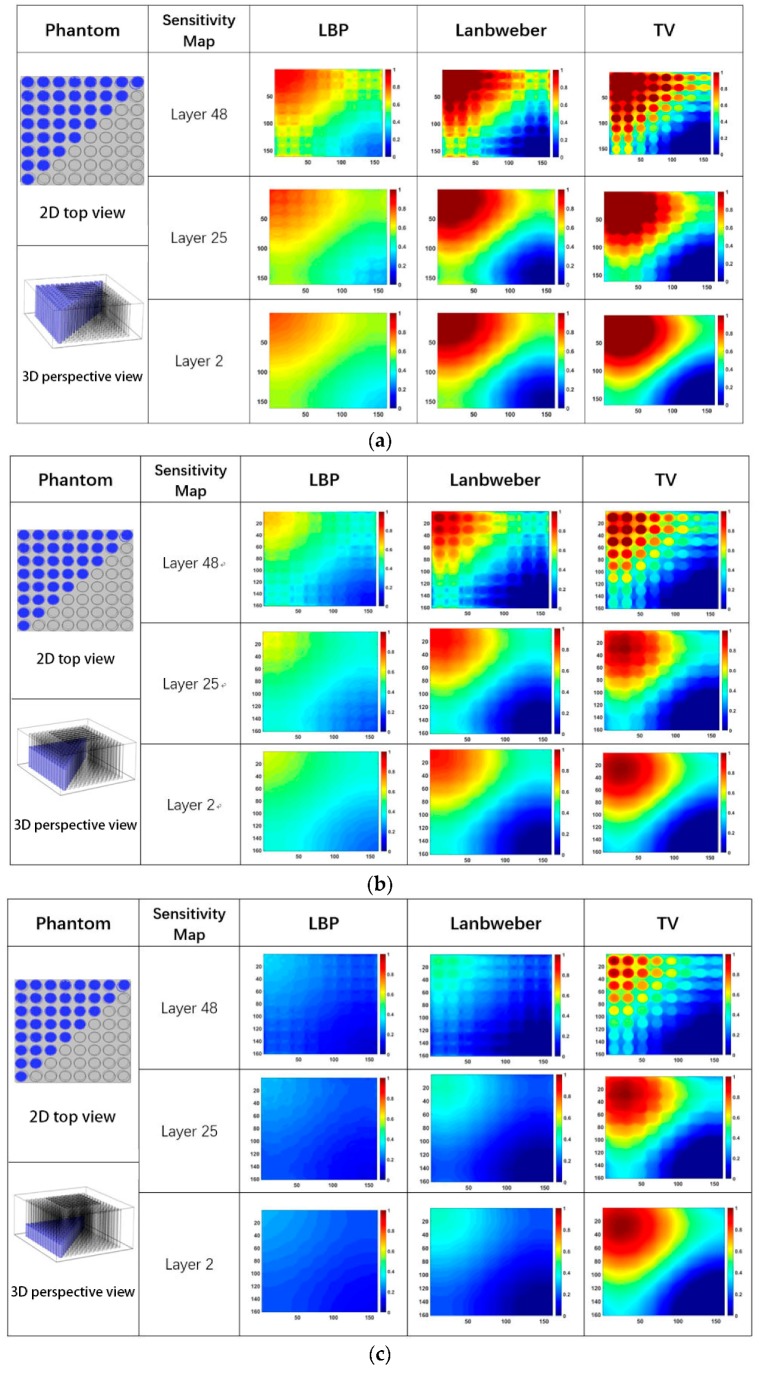
The imaging results while the column holes at the top left triangular region are full of different quantities of water, (**a**) 100% full of water, (**b**) 50% full of water, (**c**) 25% full of water.

Table 3 lists capacitances between different electrode pairs while the column holes at the top left triangular region inside the sensor are full of different quantities of water. Figure 11 shows the corresponding reconstructed images based on the measured capacitance data in Table 3 by using the LBP, the Landweber iteration, and total variation-based iteration. The phantom is illustrated by using 2D top view and 3D perspective view to make it easy to understand. Obviously, the images give larger contrast between the position of the water leakage and other areas without water leakage.

## 4. Discussion

It is worth pointing out that all results demonstrated in Section 3 are based on the measurement data obtained from our developed prototype experimental setup, which is depicted in Figure 7 and composed of an eight-electrode capacitance sensor array, an AH-2550A high-precision capacitance bridge manufactured by Andeen–Hagerling^TM^ for capacitance measuring, and a Lenovo^TM^ T440 laptop computer for image reconstruction. Compared to the preliminary results published in our previous work in [35], which are based on simulation data, these experimental data-based results in Section 3 demonstrate comprehensively that our proposed capacitance array provides qualitative imaging of water leakage in the slab. Meanwhile, quantitative imaging for evaluation of different water leakages, particularly in the region near the adhesive bonding, is still a challenge. For further study, other image reconstruction techniques, such as the level set method that may provide high-quality image reconstruction, should be developed for obtaining more quantitative results.

**Figure 12:**
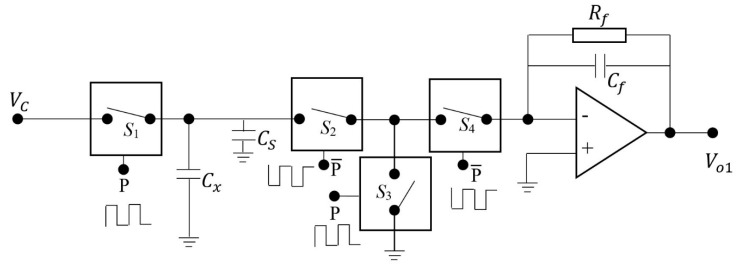
The capacitance measurement circuit for water leakage monitoring and detection.

From the point view of practical application, a measurement hardware or circuit should be designed to substitute for the commercialized capacitance bridge instrument used in our experiments. For this purpose, the most important issue is that the big electrode, the hull of submarine functioning as the common electrode of the proposed capacitance sensor array, should be considered being grounded in practice. In other words, one electrode of the capacitance to be measured is always connected to the ground. Referring to the measurement circuit proposed by Huang et al. for electrical capacitance tomography [22,23], we designed a circuit, shown in Figure 12, to implement the capacitance measurement of the proposed capacitance sensor array for water leakage detection.

In Figure 12, $C_{x}$ is the capacitance to be measured, with one electrode grounded during the whole measurement procedure. $C_{s}$ is the stray capacitance connected in parallel to $C_{x}$. By controlling the electronic switches S_1_, S_2_, S_3_, and S4 to be ’on’ and ’off’ in a proper sequence, $C_{x}$ and $C_{s}$ will be charged first and then discharge through the detection amplifier and lead to an output that is dependent on $C_{x}$ and $C_{s}$. S1 and S3 are controlled by the signal P. When P is ’1′, S1 and S3 are switched to ’on’, and when P is ’0′, S1 and S3 are switched to ’off’. The working states of S2 and S4 are opposite to S1 and S3, which are controlled by $\bar{P}$ (i.e., the reverse of P). When $\bar{P}$ is ’1′, S2 and S4 are switched to ’on’, and when $\bar{P}$ is ’0′, S2 and S4 are switched to ’off’. The frequency of P is charging and discharge frequency. According to [22], the output of the circuit (i.e., $V_{o1}$) is proportional to the charging and discharge frequency f, the voltage $V_{c}$ and the feedback resistance $R_{f}$, while the condition of $fR_{f}C_{f}>>1$ is fulfilled. That is,

(7) $$V_{o1}=-fV_{c}R_{f}\left(C_{x}+C_{s}\right)$$

**Figure 13:**
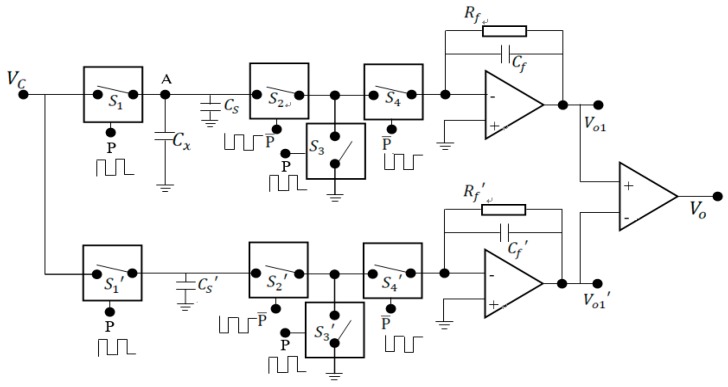
A capacitance measurement circuit with differential output for water leakage monitoring and detection.

**Figure 14:**
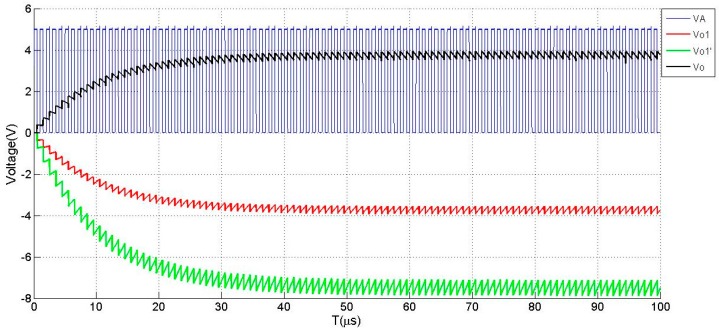
Simulated output of the differential charging and discharging circuit in Figure 14.

In comparison to the original reported circuit in [22,23], it is worth noting that the stray capacitance $C_{s}$ in the circuit depicted in Figure 13 will have an effect on the output of the measurement circuit. It is very important that $C_{s}$ should be as small as possible while doing the printed circuit board (PCB) design in practice. For this purpose, a measurement circuit with differential output to suppress the effect of $C_{s}$ is proposed and depicted in Figure 13. While laying out the PCB, another channel with the same structure as the measurement circuit depicted in Figure 12 will be duplicated, which is supposed to measure only the stray capacitance $C_{s}{}^{\prime }$. To eliminate the effect of stray capacitance $C_{s}$, the value of $C_{s}{}^{\prime }$ is theoretically kept as close as possible to the value of $C_{s}$ by duplicating the layout of measurement channel. With the continuation of charge and discharge, the output of the upper and lower branches reaches a steady state. Figure 14 depicts the simulated output of the differential circuit when $C_{s}{}^{\prime }=30\;\mathrm{pF}$ and $C_{s}=10\;\mathrm{pF}$, $C_{x}=5\;\mathrm{pF}$.

**Figure 15:**
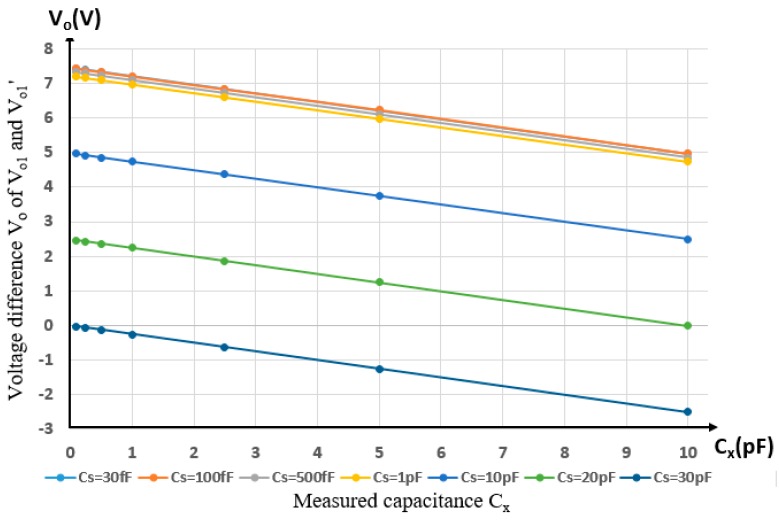
The output $V_{o}$ under different $C_{x}$ and $C_{s}$.

Table 4 and Figure 15 depict the characteristics of output $V_{o}$ with respect to the change of $C_{x}$, $C_{s}$, and $C_{s}{}^{\prime }$. It is found that the output $V_{o}$ demonstrates a perfect linear relationship with the capacitance $C_{x}$ to be measured. Furthermore, Figure 15 shows that choosing the value of $C_{s}{}^{\prime }$ as close as possible to the value of $C_{s}$ by duplicating the layout of measurement channel is effectively compensating the effect of stray capacitance $C_{s}$.

Future implementation of the hardware related to the aforementioned scheme depicted in Figure 13 may be considered with FPGA-based techniques [46].

## 5. Conclusions

In this paper, an electrical capacitance array for detecting and imaging of water leakage inside insulating slabs with porous cells is presented. The finite element method is used to characterize the sensitivity distribution of the proposed capacitance sensor array. A test setup based on commercialized capacitance bridge instrument is developed to carry out a series of test experiments. The linear back projection, the projected Landweber iteration, and the total variation regularization are used to reconstruct images from the experimental data obtained from the test setup, which are corresponding to different water leakage cases. These preliminary experimental results demonstrate that the proposed capacitance array is for providing qualitative contrast images standing for water leakage position and relative quantity. Future studies will focus on the development of new image reconstruction algorithms to obtain more quantitative results with high image quality. Another issue about the consistency of the performance of the proposed method under the condition of saline water will also be explored.

## Figures and Tables

**Table 1:** The measured capacitances between different electrode pairs while the central column hole is full of different quantities of water.

Electrode Pairs	Capacitance while All Column Holes Have No Water Inside (pF)		Capacitance while All Column Holes Are Full of Water (pF)		Capacitance while the Central Four Column Holes are 100% Full of Water (pF)		Capacitance while the Central Four Column Holes are 50% Full of Water (pF)		Capacitance while the Central Four Column Holes are 25% Full of Water (pF)	
	Horizontal Capacitance	Vertical Capacitance	Horizontal Capacitance	Vertical Capacitance	Horizontal Capacitance	Vertical Capacitance	Horizontal Capacitance	Vertical Capacitance	Horizontal Capacitance	Vertical Capacitance
E1-Common	0.336	0.351	4.315	4.361	0.357	0.373	0.369	0.381	0.346	0.359
E2-Common	0355	0.395	4.354	4.405	0.427	0.452	0.444	0.478	0.387	0.428
E3-Common	0.375	0.437	4.377	4.462	0.649	0.731	0.579	0.640	0.442	0.504
E4-Common	0.394	0.446	4.362	4.366	1.291	1.346	0.781	0.828	0.523	0.572
E5-Common	0.419	0.421	4.388	4.417	1.319	1.341	0.805	0.812	0.548	0.549
E6-Common	0.449	0.392	4.315	4.351	0.729	0.688	0.648	0.586	0.514	0.455
E7-Common	0.450	0.367	4.329	4.318	0.501	0.454	0.523	0.446	0.482	0.396
E8-Common	0.397	0.336	4.372	4.287	0.421	0.352	0.426	0.367	0.405	0.345

**Table 2:** The measured capacitances between different electrode pairs while two groups of column holes at two different places on one side of the slab are full of different quantities of water.

Electrode Pairs	Capacitance While All Column Holes Have No Water Inside (pF)		Capacitance While All Column Holes are Full of Water (pF)		Capacitance While Two Groups of Column Holes at Two Different Places on One Side of the Slab Are 100% Full of Water (pF)		Capacitance While Two Groups of Column Holes at Two Different Places on One Side of the Slab are 50% Full of Water (pF)		Capacitance While Two Groups of Column Holes at Two Different Places on One Side of the Slab are 25% Full of Water (pF)	
	Horizontal Capacitance Array	Vertical Capacitance Array	Horizontal Capacitance Array	Vertical Capacitance Array	Horizontal Capacitance Array	Vertical Capacitance Array	Horizontal Capacitance Array	Vertical Capacitance Array	Horizontal Capacitance Array	Vertical Capacitance Array
E1-Common	0.336	0.351	4.315	4.361	0.612	0.387	0.585	0.320	0.545	0.321
E2-Common	0355	0.395	4.354	4.405	1.326	0.423	1.169	0.347	0.943	0.342
E3-Common	0.375	0.437	4.377	4.462	1.328	0.463	1.192	0.375	0.940	0.359
E4-Common	0.394	0.446	4.362	4.366	0.695	0.494	0.612	0.426	0.551	0.409
E5-Common	0.419	0.421	4.388	4.417	0.695	0.861	0.615	0.761	0.556	0.709
E6-Common	0.449	0.392	4.315	4.351	1.337	2.015	1.130	1.837	1.006	1.549
E7-Common	0.450	0.367	4.329	4.318	1.331	2.002	1.131	1.841	0.998	1.540
E8-Common	0.397	0.336	4.372	4.287	0.658	0.935	0.549	0.874	0.514	0.771

**Table 3:** The measured capacitances between different electrode pairs while one triangular region is full of different quantities of water.

Electrode Pairs	Capacitance While All Column Holes Have No Water Inside (pF)		Capacitance While All Column Holes are Full of Water (pF)		Capacitance While Column Holes in Top Left Triangular Region are 100% Full of Water (pF)		Capacitance While Column Holes in Top Left Triangular Region are 50% Full of Water (pF)		Capacitance While Column Holes in Top Left Triangular Region Are 25% Full of Water (pF)	
	Horizontal Capacitance Array	Vertical Capacitance Array	Horizontal Capacitance Array	Vertical Capacitance Array	Horizontal Capacitance Array	Vertical Capacitance Array	Horizontal Capacitance Array	Vertical Capacitance Array	Horizontal Capacitance Array	Vertical Capacitance Array
E1-Common	0.336	0.351	4.315	4.361	4.122	4.075	3.338	3.382	1.946	1.892
E2-Common	0355	0.395	4.354	4.405	3.822	3.731	3.059	2.991	1.764	1.708
E3-Common	0.375	0.437	4.377	4.462	3.408	3.380	2.742	2.705	1.527	1.502
E4-Common	0.394	0.446	4.362	4.366	3.012	2.966	2.364	2.372	1.296	1.292
E5-Common	0.419	0.421	4.388	4.417	2.659	2.453	2.002	2.044	1.083	1.031
E6-Common	0.449	0.392	4.315	4.351	2.209	2.099	1.665	1.675	0.885	0.819
E7-Common	0.450	0.367	4.329	4.318	1.680	1.584	1.249	1.216	0.693	0.599
E8-Common	0.397	0.336	4.372	4.287	1.246	1.162	0.888	0.848	0.549	0.426

**Table 4:** The output voltage $V_{o}$ under different $C_{x}$ and $C_{s}$.

C_x_ (pF)	V_o_ (V) under Different Cs and Fixed Cs’ = 30 pF						
	Cs = 30 fF	Cs = 100 fF	Cs = 500 fF	Cs = 1 pF	Cs = 10 pF	Cs = 20 pF	Cs = 30 pF
**10**	4.978	4.962	4.861	4.735	2.493	0	−2.495
**5**	6.223	6.23	6.106	5.982	3.742	1.246	−1.245
**2.5**	6.843	6.827	6.726	6.603	4.365	1.872	−0.623
**1**	7.215	7.197	7.099	6.975	4.735	2.245	−0.249
**0.5**	7.339	7.322	7.223	7.099	4.861	2.37	−0.124
**0.25**	7.399	7.382	7.285	7.159	4.925	2.431	−0.065
**0.1**	7.420	7.420	7.322	7.197	4.962	2.469	−0.029
